# Multivariable Prediction Model for Suspected Ocular Myasthenia Gravis: Development and Validation

**DOI:** 10.1097/WNO.0000000000002346

**Published:** 2025-04-21

**Authors:** Armin Handzic, Marius P. Furter, Brigitte C. Messmer, Magdalena A. Wirth, Yulia Valko, Fabienne C. Fierz, Edward A. Margolin, Konrad P. Weber

**Affiliations:** Department of Ophthalmology (AH, BCM, MAW, FCF, KPW), University Hospital Zurich, University of Zurich, Zurich, Switzerland; University of Toronto (AH, EAM), Faculty of Medicine, Department of Ophthalmology and Vision Sciences, Toronto, Ontario, Canada; Institute for Mathematics (IMATH) (MPF), University of Zurich, Zurich, Switzerland; Department of Neurology (YV, KPW), University Hospital Zurich, University of Zurich, Zurich, Switzerland; and Division of Neurology, Department of Medicine (EAM), Faculty of Medicine, University of Toronto, Toronto, Ontario, Canada.

## Abstract

**Background:**

Diagnosing ocular myasthenia gravis (OMG) remains challenging despite recent diagnostic advances. We addressed this challenge by developing and validating a multivariable prediction model that estimates the OMG probability given the results of any partial selection of available diagnostic tests.

**Methods:**

The source data for our model were retrieved from our blinded prospective diagnostic accuracy study at the University Hospital Zurich (USZ). Patients with ptosis and/or diplopia whose presentation was suspicious for OMG underwent comprehensive diagnostic testing. An independent neuromuscular specialist made the final diagnosis. These data were used to fit and validate a Bayesian network model against additional retrospective USZ and the University of Toronto (UoT) patient data. The primary outcome was to predict the likelihood of a positive OMG diagnosis given the available diagnostic tests. For any set of tests, the model returns an OMG probability together with 95% credible intervals, indicating the prediction uncertainty.

**Results:**

Of 89 patients included in the development of the model, 39 were diagnosed with OMG. Based on our Bayesian network model, the following variables were the most useful predictors in descending order: edrophonium test, acetylcholine receptor (AChR) antibodies), single-fiber electromyogram (sfEMG), repetitive nerve stimulations (RNS) facial nerve, RNS accessory nerve, Besinger score, ice test, sustained upgaze test, dysarthria, dyspnea, dysphagia, diplopia, ptosis, age, and sex. The model was validated by determining the mean error rate and the area under the curve (AUC) by both 10-fold cross-validation and prediction on the retrospective USZ and UoT validation data consisting of 69 and 24 patients, respectively. Of all variables, edrophonium (sensitivity 94%, specificity 90%) and AChR antibody testing (sensitivity 85%, specificity 96%) showed the highest predictive value during validation with an AUC of 0.912 and 0.872, respectively. Incorporating more predictors reduced the predictive error in both validation data sets.

**Conclusions:**

Our prediction model serves as a basis to predict the OMG likelihood. It underwent successful internal and external validation and can be used to assist in clinical decision making.

## Abstract 2

Supplemental Digital Content is Available in the Text.

Diagnosing ocular myasthenia gravis (OMG) remains a clinical challenge despite recent diagnostic advances. It is a great mimicker of many ocular motility disorders, and acetylcholine receptor (AChR) antibody testing, its most accessible and specific diagnostic biomarker, only demonstrates a sensitivity of approximately 39%–71%.^1–5^ Further ancillary testing, such as the edrophonium test, or electrophysiologic examinations (repetitive nerve stimulation (RNS) and single-fiber electromyography (sfEMG) are often difficult to access and require experienced examiners.^6^

OMG's clinical hallmark is fluctuating fatigable and usually reversible muscle weakness. Hence, the clinical diagnosis of OMG is challenging due to its variable and undulating symptoms. Approximately 80% of myasthenia patients first become symptomatic with isolated diplopia or ptosis and 50% generalize within 2 years.^7^ Delayed diagnosis of this treatable disorder may lead to preventable life-threatening complications.^8^ Thus, early diagnosis is paramount not only for ophthalmologists and neurologists but also for emergency physicians alike.

However, approximately 50% of patients do not receive the correct diagnosis within the first year of symptoms.^9^ Therefore, we aimed to address this by developing and validating a multivariable prediction model to estimate OMG probability based on any subset of available tests. Our model should be applicable in clinical practice to support the diagnosis of seronegative OMG.

## METHODS

### Source Data

The data were retrieved from our blinded prospective diagnostic accuracy study at the Departments of Ophthalmology and Neurology, University Hospital Zurich (USZ), from November 2016 to December 2019 (ClinicalTrials.gov NCT03049956).^10^ Informed written consent was obtained from all participants. We developed the multivariable predictive model using this cohort, following TRIPOD+AI guidelines.^11,12^ The studies were approved by the local ethics committees of Zurich (BASEC-Nr.2016-01109) and Toronto (Nr.44862) and adhered to the Declaration of Helsinki. Additional retrospective data of suspected OMG patients from USZ and a tertiary-level practice affiliated with the University of Toronto (UoT) from 2023 were included to validate the model.

### Participants

Data from 89 adults from our previous prospective diagnostic accuracy study with ptosis and/or diplopia suspicious of OMG were included.^10^ The goal of this study was to test the diagnostic accuracy of repetitive ocular vestibular evoked myogenic potentials (roVEMP) to detect a decrement directly in the extraocular muscle activity as a novel test for the diagnosis of OMG. For this purpose, the patients underwent extensive diagnostic testing, including sustained-upgaze-test, ice pack-test, Besinger score, serum autoantibodies (AChR, anti-Titin, anti-MuSK, and anti-LRP4), edrophonium testing, RNS, and sfEMG as a reference standard for th OMG diagnosis. The final diagnosis was made by a neuromuscular specialist after three-month follow-up, including evaluation of any treatment response.

OMG diagnosis in the retrospective USZ and UoT cohorts was established by experienced neuro-ophthalmologists (K.P.W., E.A.M.) by using all available data. Retrospective data from 69 USZ and 24 UoT patients, including patients with a refuted OMG diagnosis, were used for external validation. The final diagnosis of OMG was made in 39, 42, and 23 cases in the prospective USZ, retrospective USZ, and UoT cohorts, respectively.

### Outcome

The multivariable model was designed to predict the likelihood of OMG diagnosis given the available diagnostic tests and the patient's sex and age. The outcome assessment was blinded as our biostatistician (M.P.F.) was not involved in data collection or involved in reaching a diagnosis.

### Predictors

Model predictors (Table 1) were age, sex, ptosis, diplopia, sustained upgaze test, ice pack test, AChR antibodies, Besinger score, edrophonium test, RNS, and sfEMG. The continuous variables, “age” and “Besinger score,” were binned into discrete categories. RNS measurements were performed twice on both facial and accessory nerves and were considered positive if any measurement was abnormal. The predictors were chosen based on clinical experience and feasibility.^13,14^

**TABLE 1.** Detailed counts for all model variables across the training and validation data sets for patients suspected of having OMG

	OMG	Study			USZ			UoT		
		Negative (n = 50)	Positive (n = 39)	Total (n = 89)	Negative (n = 27)	Positive (n = 42)	Total (n = 69)	Negative (n = 1)	Positive (n = 23)	Total (n = 24)
Age	(0–50) (50–70) (70–120)	15 17 18	10 13 16	25 30 34	5 14 8	8 16 18	13 30 26	0 1 0	3 13 7	3 14 7
Sex	Male Female	18 32	23 16	41 48	13 14	30 12	43 26	1 0	13 10	14 10
Diplopia	Negative Positive	14 36	15 24	29 60	6 21	7 35	13 56	0 1	4 19	4 20
Ptosis	Negative Positive	19 31	10 29	29 60	10 17	14 28	24 45	0 1	4 19	4 20
AChR antibody	Negative Positive NA	48 2 0	6 33 0	54 35 0	26 0 1	10 32 0	36 32 1	1 0 0	7 16 0	8 16 0
Sustained upgaze test	Negative Positive NA	32 14 4	13 25 1	45 39 5	17 9 1	14 27 1	31 36 2	0 1 0	1 10 12	1 11 12
Ice test	Negative Positive NA	21 9 20	8 13 18	29 22 38	2 4 21	4 4 34	6 8 55	0 0 1	0 2 21	0 2 22
Besinger/QMG score	(−1 to 1) (1–4) (4–8) (8–24) NA	5 25 18 2 0	5 14 16 4 0	10 39 34 6 0	0 1 3 0 23	1 9 6 12 14	1 10 9 12 37			
Edrophonium	Negative Positive NA	36 4 10	2 29 8	38 33 18	12 1 14	5 13 24	17 14 38			
RNS	Negative Positive NA	42 8 0	14 23 2	56 31 2	7 0 20	5 1 36	12 1 56			
sfEMG	Negative Positive NA	28 22 0	9 27 3	37 49 3	0 1 26	0 1 41	0 2 67			

In the USZ validation data, the QMG score was measured instead of the Besinger score.

AChR antibody, acetylcholine receptor antibody; OMG, ocular myasthenia gravis; QMG, quantitative myasthenia gravis score; RNS, combined results from repetitive nerve stimulation of accessory and facial nerve; sfEMG, single-fibre electromyography; Study, prospective cohort from the University Hospital Zurich; USZ, retrospective cohort from the University Hospital Zurich; UoT, retrospective cohort from the University of Toronto.

### Missing Data

The model was fitted using the complete cases of the prospective data set. Thus, no data were imputed. The retrospective USZ and UoT data were allowed to have missing examinations, in line with the model's purpose for use in clinics.

### Statistical Analysis

The study data were used to fit a discrete Bayesian network model^15,16^ using the R package bnlearn^17^ (supplemental file “model_fit.R”). In contrast to regression models, Bayesian networks represent a joint distribution over all variables. Consequently, by conditioning the values of any subset of variables, one can predict the probability of the remaining ones.^15^ This allows the model to predict the OMG probability based on any test combination, even with missing examinations. Bayesian networks construct their joint distribution from a set of simpler conditional probability distributions (CPDs) according to a directed acyclic graph (DAG, See **Supplemental Digital Content**, Appendix 1, http://links.lww.com/WNO/A936).

### Internal Model Validation

The fitted model was internally validated by 10-fold cross-validation based on the error rate (percentage of wrong classifications) and area under the receiver operator characteristic curve (AUC). Because the error rate depends on the chosen set of predictors, the validation was performed on several representative predictor sets. A prediction was considered positive if the median probability of OMG, given the considered predictors, was >50%. Only complete data sets with respect to the selected predictors were considered.

### External Model Validation

External model validations were performed with separate retrospective USZ and UoT data using the same criteria as for the internal validation. Because these external data were obtained from clinical practice, they contained incomplete data sets with fewer diagnostic tests than the study data for the model. Hence, these data could only be used to evaluate the model performance on the most common diagnostic predictors.

## RESULTS

This multivariable prediction model was trained on the prospective study data^10^ and validated against retrospective data from USZ and UoT. Table 1 summarizes the cases for each predictor variable across the 3 data sets. The training data included 89 patients of which 39 (44%) diagnosed with OMG. The USZ validation data included 69 patients with 42 OMG cases (61%), whereas the UoT comprised 24 patients, with 23 OMG patients (96%). Note that there were missing values in all 3 data sets. Moreover, the UoT data sets did not include Besinger/QMG scores, edrophonium test, RNS, or sfEMG.

The following variables were the most useful predictors in descending order: edrophonium test, AChR antibodies, sfEMG, RNS facial/accessory nerve, Besinger score, ice test, sustained upgaze test, dysarthria, dyspnea, dysphagia, diplopia, ptosis, age, and sex. The sensitivity and specificity of each diagnostic test for OMG was determined from the counts in Table 1. As expected, edrophonium testing was highly sensitive (29/31 = 94%) and specific (36/40 = 90%) in the prospective USZ training data. The USZ validation data showed a sensitivity of 72% (13/18) and a specificity of 92% (12/13). However, in the USZ validation data, the edrophonium test was only performed in 31 of 69 cases. The anti-AChR antibody test also displayed high sensitivity (33/39 = 85%) and specificity (48/50 = 96%) in the training data. Interestingly, the sensitivity among 18- to 50-year old patients was markedly lower (6/10 = 60%) than for those older than 50 years (27/29 = 93%). The sensitivity was also lower in the validation data (USZ:32/42 = 76%, UoT:16/23 = 70%) but still significantly above the 40% and 55% reported in other studies.^1,2^ The accuracy of the other tests were much lower. In the training set, the sustained upgaze test had a sensitivity of 66% (25/38) and a specificity of 70% (32/46). Similarly, sfEMG showed a sensitivity of 75% (27/36) with a specificity of 56% (28/50).

An online version of our Bayesian model can be accessed online: https://myasthenia-prediction.app/(without individual patient data). Physicians can enter the known clinical parameters in the appropriate fields, and the estimated median OMG probability will be displayed with 95% credible intervals (CI). The prediction uncertainty is visible in 2 ways. First, a higher probability indicates that an OMG diagnosis is more likely. Second, a tighter CI around the expected value means the predicted value is more certain. Our model is designed in a way that a prediction is possible even with missing clinical parameters. The full model is accessible online theough the GitHub developer platform https://github.com/MariusFurter/myasthenia-prediction/and archived in the EU Open Research Repository Zenodo.^18^

The model's learned CPDs are presented in Table 2 as 95% CIs around the median. For example, the median probability of a positive edrophonium test, given that the patient has OMG, was learned to be 91.7%. Moreover, it is 95% certain that this probability lies between 79% and 98%. The corresponding 95% CI is represented as (79.0, 91.7%, 98.0) in the table.

**TABLE 2.** Conditional probability distribution tables for all model variables (%) for patients suspected of having OMG

Age distribution	***18–50 y***	***50–70 y***	***70 y+***
	19.4, **28.1%**, 37.7	24.4, **33.7%**, 43.8	28.2, **37.9%**, 48.5
Sex ratio	*Male*	*Female*	—
	36.2, **46.1%**, 56.4	43.6, **53.9%**, 63.8	
OMG		*Negative*	*Positive*
	Sex		
	Male	30.0, **44.0%**, 59.2	40.8, **56.0%**, 70.0
	Female	52.5, **66.2%**, 78.4	21.6, **33.8%**, 47.5
AChR antibodies		*Negative*	*Positive*
OMG	Age		
*Negative*	18–50 y	79.2, **95.8%**, 99.9	0.148, **4.17%**, 20.8
	50–70 y	81.3, **96.2%**, 99.9	0.14, **3.77%**, 18.7
	70 y+	66.8, **86.1%**, 96.6	3.41, **13.9%**, 33.2
*Positive*	18–50 y	16.7, **41.5%**, 69.4	30.6, **58.5%**, 83.3
	50–70 y	1.71, **11.6%**, 33.7	66.3, **88.4%**, 98.3
	70 y+	1.44, **9.74%**, 28.5	71.5, **90.3%**, 98.6
Edrophonium		*Negative*	*Positive*
	OMG		
	*Negative*	76.8, **88.6%**, 95.9	4.07, **11.4%**, 23.2
	*Positive*	2.02, **8.33%**, 21.0	79.0, **91.7%**, 98.0
Sustained upgaze test		*Negative*	*Positive*
	OMG		
	*Negative*	54.9, **68.9%**, 80.9	19.1, **31.1%**, 45.1
	*Positive*	21.0, **34.6%**, 50.5	49.5, **65.4%**, 79.0
Ice test	OMG	*Negative*	*Positive*
	*Negative*	52.0, **69.2%**, 83.4	16.6, **30.8%**, 48.0
	*Positive*	20.8, **38.7%**, 59.3	40.7, **61.3%**, 79.2
sfEMG		*Negative*	*Positive*
	OMG		
	*Negative*	42.3, **56.0%**, 68.9	31.1, **44.0%**, 57.7
	*Positive*	13.8, **25.9%**, 41.3	58.7, **74.1%**, 86.2
RNS		*Negative*	*Positive*
	OMG		
	*Negative*	71.3, **83.1%**, 91.5	8.46, **16.9%**, 28.7
	*Positive*	24.0, **38.2%**, 54.1	45.9, **61.8%**, 76.0

Besinger score		*(0,1)*	*(1,4)*	*(4,8)*	*(8,24)*
OMG	Age				
*Negative*	18–50 y	9.82, **25.5%**, 47.9	30.8, **52.8%**, 74.1	3.67, **14.6%**, 34.6	0.13, **3.76%**, 18.3
	50–70 y	0.105, **3.39%**, 16.8	23.3, **42.8%**, 63.9	18.9, **37.6%**, 59.3	3.10, **13.1%**, 31.4
	70 y+	1.19, **7.87%**, 23.5	22.0, **40.5%**, 61.9	25.5, **45.4%**, 66.2	0.117, **3.23%**, 16.2
*Positive*	18–50 y	9.21, **27.5%**, 53.6	8.73, **27.6%**, 53.8	9.01, **27.5%**, 53.1	1.95, **12.6%**, 36.7
	50–70 y	1.58, **10.3%**, 30.4	24.2, **46.7%**, 69.7	10.9, **28.7%**, 52.2	1.54, **10.4%**, 30.0
	70 y+	1.23, **8.64%**, 26.7	9.10, **24.3%**, 45.8	28.4, **49.7%**, 71.0	3.37, **13.7%**, 33.4

Diplopia		*Negative*	*Positive*
OMG	Age		
*Negative*	*18–50 y*	29.5, **53.1%**, 74.9	25.1, **46.9%**, 70.5
	50–70 y	9.50, **25.5%**, 47.8	52.2, **74.5%**, 90.5
	*70 y+*	3.30, **14.0%**, 33.3	66.7, **86.0%**, 96.7
*Positive*	*18–50 y*	11.3, **32.3%**, 60.3	39.7, **67.7%**, 88.7
	*50–70 y*	12.9, **32.4%**, 57.4	42.6, **67.6%**, 87.1
	*70 y+*	28.3, **50.3%**, 72.0	28.0, **49.7%**, 71.7

Ptosis		*Negative*	*Positive*
OMG	Diplopia		
*Negative*	*Negative*	26.4, **50.0%**, 73.6	26.4, **50.0%**, 73.6
	*Positive*	19.9, **34.0%**, 49.6	50.4, **66.0%**, 80.1
*Positive*	*Negative*	15.0, **34.3%**, 58.8	41.2, **65.7%**, 85.0
	*Positive*	9.54, **22.4%**, 40.9	59.1, **77.6%**, 90.5

Each column (except the first 2) represents a CPD p (X | pa(X)) for the variable X indicated in the top row. The possible values for the parents pa (X) are listed in the leftmost columns. The 3 numbers in each CPD entry summarize a 95% credible interval around the posterior median in the form (0.025 quantile, median, 0.975 quantile). For instance, in the column for OMG, the entry on the top left represents a 95% credible interval for the probability of OMG being negative, given that the patient is male.

AChR antibody, acetylcholine receptor antibody; OMG, ocular myasthenia gravis; RNS, combined results from repetitive nerve stimulation of accessory and facial nerve; sfEMG, single-fibre electromyography.

The model was validated by determining the mean error rate and AUC for several representative predictor sets by 10-fold cross-validation and prediction on the retrospective USZ and UoT validation data (Table 3). A case was classified as OMG positive if the predicted probability was >50%. Note that with this approach, the uncertainty information in the CI, which is also a model output, is lost. Hence, the model could have practical value even for variables with high error rates by indicating the diagnostic uncertainty.

**TABLE 3.** Mean error rates and mean area under operator receiver operating characteristic curve (AUC) for selected predictor sets for training data, 10-fold cross-validation and external validation on the retrospective USZ and UoT test sets for patients suspected of having OMG

Variables	Training Data			10-Fold Cross-Validation			USZ			UoT		
	Cases	Mean Error	Mean AUC	Cases	Mean Error	Mean AUC	Cases	Mean Error	Mean AUC	Cases	Mean Error	Mean AUC
Age, sex	89	39.3%	0.612	89	41.9%	0.64	69	40.9%	0.612	24	61.9%	0.614
Diplopia, ptosis	89	43.5%	0.576	89	49.4%	0.527	69	55.7%	0.496	24	73.5%	0.503
AChR antibodies	89	8.99%	0.906	89	9.2%	0.872	68	14.7%	0.874	24	29.2%	0.879
Sustained upgaze test	84	32.9%	0.678	84	33.9%	0.628	67	35.5%	0.659	12	19.6%	0.655
Ice test	51	34.5%	0.660	51	32.2%	0.729	14	57.0%	0.424	—	—	—
Besinger score	89	43.6%	0.561	89	47.1%	0.494	32	55.3%	0.551	—	—	—
Edrophonium	71	8.45%	0.915	71	8.4%	0.912	31	19.4%	0.821	—	—	—
RNS	87	25.4%	0.732	87	25.2%	0.767	13	38.6%	0.571	—	—	—
sfEMG	86	37.1%	0.653	86	39.4%	0.689	—	—	—	—	—	—
Edrophonium, AChR antibodies	71	7.86%	0.975	71	9.1%	0.979	30	20.4%	0.897	—	—	—
Age, sex, diplopia, ptosis, Sustained upgaze test	84	31.0%	0.735	84	34.0%	0.707	67	40.1%	0.65	12	34.5%	0.65
Age, sex, diplopia, ptosis, Sustained upgaze test, AChR antibodies	84	12.5%	0.919	84	14.9%	0.834	66	15.8%	0.909	12	25.0%	0.909

The number of cases that could be used for validation is indicated.

AChR antibody, acetylcholine receptor antibody; QMG, quantitative myasthenia gravis score; RNS, combined results from repetitive nerve stimulation of accessory and facial nerve; sfEMG, single-fibre electromyography; USZ, retrospective cohort from the University Hospital Zurich; UoT, retrospective cohort from the University of Toronto.

In cross-validation, both edrophonium (8.4% error, 0.912 AUC) and anti-AChR-tests (9.2%, 0.872) performed well individually in predicting OMG. The next best predictor was RNS (25.2%, 0.767), followed by the ice test (32.2%, 0.729), sustained upgaze test (33.9%, 0.628), and sfEMG (39.4%, 0.689). Knowledge of the Besinger score (47.1%, 0.494) or ptosis and diplopia (49.4%, 0.527) did not improve prediction above guessing level. This is not surprising in the case of diplopia and ptosis because these variables served as inclusion criteria.

The cross-validation results were compared with the predictive performance on the 2 external validation data sets from USZ and UoT (Table 3). Generally, the error rates and AUC in the USZ data mirror those obtained by cross-validation, although the errors were somewhat higher. For instance, edrophonium test and AChR antibody tests had predictive errors of 19.4% and 14.7%, respectively. The cases where the performance on the USZ validation data deviated from cross-validation, namely, ice test and RNS are all associated with low numbers in the validation data set and should thus be regarded with caution. To assess possible overfitting, we calculated the mean error and mean AUC on the training data (Table 3). The results closely match those obtained in 10-fold cross-validation, demonstrating that the model does not overfit to the training data.

The predictive errors on the UoT data were much higher than in cross-validation (Table 3). Even AChR antibody testing showed 29.2% error rate. However, the UoT data were a significantly smaller and biased sample with 23/24 OMG-positive patients. By contrast, the AUC scores, which are independent of the available number of clinical variables, were remarkably stable across all validation approaches.

## DISCUSSION

We developed a Bayesian prediction model^18^ based on a prospective cohort of patients with suspected OMG who rigorously underwent all available diagnostic modalities.^10^ The model underwent internal and external validation by both 10-fold cross-validation and prediction on retrospective USZ and UoT validation data. Moreover, it has a fully comprehensible algorithm and a high level of flexibility to deal with missing variables. This model is intended to be used online in clinical settings with limited diagnostic resources to assist in diagnosing seronegative OMG (https://myasthenia-prediction.app/).

To achieve the goal of an adaptive model, we had to overcome the obstacle of missing clinical examinations, as not all tests are usually done in clinical practice. This was accomplished by jointly modeling all variables using a Bayesian network, which allows predicting the OMG probability even with incomplete diagnostic workup. Therefore, the model should be widely available to clinicians, who may not have access to sophisticated ancillary testing. The model can also be helpful because sfEMG results may be prone to significant interoperator variability, and studies have reported sensitivity and specificity rates between 62% to 99% and 66%–98%, respectively.^5,19^ Furthermore, contraindications may prohibit certain diagnostic procedures, such as the edrophonium test, in case of pulmonary or cardiac comorbidities. Accordingly, in our retrospective USZ cohort, edrophonium testing was only performed in 31 of 69 cases due to contraindications or AChR antibody seropositivity, which likely explains the lower sensitivity of 72% compared with the more comprehensive prospective USZ cohort with a sensitivity of 94%. Regarding the sensitivity of AChR antibody testing, we found that the sensitivity in patients below 50 years of age was significantly lower (60%) than in those over 50 years (93%) - a finding consistent with Peeler et al3 who reported that the mean age of patients with positive AChR antibodies was significantly higher than those with negative test results (61.2 years vs 54.7 years). The sensitivity was also lower in the validation data (USZ: 76%, UoT: 70%) but still above the 40% and 55% reported in other studies.^1,2^

In our model, diplopia and ptosis did not have strong predictive values for diagnosing OMG. Thus, more specific predictors, such as fatiguability of ptosis or variable diplopia patterns should be used to refine the model further. Encouragingly, including more predictors seemed to aid generalization beyond the training data as seen in the external validation results. The external Toronto data set showed higher error rates for several predictors. This likely reflects the fact that ancillary testing, such as the edrophonium test and electrophysiological testing, was not frequently performed. Thus, the diagnosis of seronegative OMG was made more frequently based on treatment response. However, we believe this represents a common real-world scenario and is also represented in the retrospective USZ cohort to a lesser degree. Importantly, all but 1 case in the UoT data was diagnosed with OMG, representing a significantly biased sample compared with the USZ cohort. This also explains why the sustained upgaze test shows a surprisingly low error of 19.6% in the UoT data. But again, using several predictors produced estimates with lower error and higher AUC in both validation data sets.

Other studies developed and validated prediction models to assess the likelihood of OMG generalization^13^ or short-term outcomes for myasthenia gravis in general.^20^ Although the first study used an accelerated failure time model, the latter study tested 14 different machine learning algorithms to find the best-fitting model, which was a random forest algorithm in their case. This leads us to the perils of the increasingly popular machine learning or artificial intelligence-based models, which ultimately remain a black box for the user. By contrast, we aimed to derive our model from medically plausible relationships between the variables. For this purpose, our Bayesian network model is based on a DAG (directed acyclic graph) (See **Supplemental Digital Content**, Figure 1, http://links.lww.com/WNO/A936) and allows for a direct evaluation of the model fit based on the obtained conditional probabilities. We believe that the comprehensibility of a prediction model is essential for its acceptance and trustworthiness in clinical practice.

A strength of our model is its development from a data set of patients with a rigorous prospective workup. Consequently, there were only very few missing data, this was likely due to a specific contraindication to performing a diagnostic test.

Some limitations in this study need to be addressed. First, given the relative rarity of OMG and the study's prospective nature, our model's sample size was relatively small. Thus, to obtain more robust and generalizable data, our model needs multicentric collaboration to expand its data set. However, our model was primarily developed as a proof of concept to find a mathematical solution for a model that could be useful in a clinical setting, even with missing examinations. Second, although some predictors representing symptoms and clinical findings are robust, they are only generally defined for the model. Including further and more specific predictors, such as spontaneous fluctuation of diplopia and ptosis over time, as well as Cogan lid twitch, may further improve the accuracy of our model. Finally, because these patients were referred to a tertiary care center, our cohort was likely confounded by a referral bias.

In conclusion, we developed and validated a Bayesian prediction model based on a rigorous prospective data set that can be used as a basis to predict the likelihood of OMG. However, establishing this diagnosis still requires clinical acumen. However, it can assist in clinical decision making on whether to diagnose seronegative OMG or consider an alternative etiology. To refine our model, it needs to be underpinned with a larger data set in future studies.

STATEMENT OF AUTHORSHIP

Conception and design: A. Handzic, M. P. Furter, K. P. Weber; Acquisition of data: A. Handzic, B. C. Messmer, M. A. Wirth, Y. Valko, F. C. Fierz, E. A. Margolin, K. P. Weber; Analysis and interpretation of data: A. Handzic, M. P. Furter, K. P. Weber. Drafting the manuscript: A. Handzic, M. P. Furter; Revising the manuscript for intellectual content: B. C. Messmer, M. A. Wirth, Y. Valko, F. C. Fierz, E. A. Margolin, K. P. Weber. Final approval of the completed manuscript: A. Handzic, M. P. Furter, B. C. Messmer, M. A. Wirth, Y. Valko, F. C. Fierz, E. A. Margolin, K. P. Weber.
